# Error on the stopping power ratio of ERKODENT's mouthpiece for head and neck carbon ion radiotherapy treatment

**DOI:** 10.1002/acm2.13987

**Published:** 2023-04-05

**Authors:** Sung Hyun Lee, Takayuki Kanai, Hikaru Souda, Yuya Miyasaka, Hongbo Chai, Takuya Ono, Yoshifumi Yamazawa, Koji Suzuki, Azusa Sato, Masashi Katsumata, Takeo Iwai

**Affiliations:** ^1^ Department of Heavy Particle Medical Science, Graduate School of Medical Science Yamagata University Iidanishi Yamagata Japan; ^2^ Department of Radiation Oncology Tokyo Women's Medical University Shinjuku Tokyo Japan; ^3^ Department of Radiology Yamagata University Hospital Iidanishi Yamagata Japan; ^4^ Accelerator Engineering Corporation Inage Chiba Japan

**Keywords:** carbon ion radiotherapy, ERKODENT, hounsfield units, mouthpiece, stopping power ratio

## Abstract

The errors on the stopping power ratio (SPR) of mouthpiece samples from ERKODENT were evaluated. Erkoflex and Erkoloc‐pro from ERKODENT and samples that combined Erkoflex and Erkoloc‐pro were computed tomography (CT)‐scanned using head and neck (HN) protocol at the East Japan Heavy Ion Center (EJHIC), and the values were averaged to obtain the CT number. The integral depth dose of the Bragg curve with and without these samples was measured for 292.1, 180.9, and 118.8 MeV/u of the carbon‐ion pencil beam using an ionization chamber with concentric electrodes at the horizontal port of the EJHIC. The average value of the water equivalent length (WEL) of each sample was obtained from the difference between the range of the Bragg curve and the thickness of the sample. To calculate the difference between the theoretical and measured values, the theoretical CT number and SPR value of the sample were calculated using the stoichiometric calibration method. Compared with the Hounsfield unit (HU)‐SPR calibration curve used at the EJHIC, the SPR error on each measured and theoretical value was calculated. The WEL value of the mouthpiece sample had an error of approximately 3.5% in the HU‐SPR calibration curve. From this error, it was evaluated that for a mouthpiece with a thickness of 10 mm, a beam range error of approximately 0.4 mm can occur, and for a mouthpiece with a thickness of 30 mm, a beam range error of approximately 1 mm can occur. For a beam passing through the mouthpiece in HN treatment, it would be practical to consider a mouthpiece margin of 1 mm to avoid beam range errors if ions pass through the mouthpiece.

## 1 INTRODUCTION

In particle therapy, the computed tomography (CT) number of the human body obtained by a CT scanner is converted into a stopping power ratio (SPR) value relative to water using the Hounsfield unit (HU)‐SPR calibration curve.^1^, ^2^ The ion energy required to locate the Bragg peak at the tumor position is then determined using the integral of the SPR information along the beam path. Nevertheless, it is known that the HU‐SPR calibration curve has an error of approximately 3% or more for the material of the human body.^3^, ^4^, ^5^ In the case of non‐human materials, the SPR converted through the HU‐SPR calibration curve differs significantly from the expected value because the CT number is affected by beam hardening and the SPR depends on electron density, whereas the CT number depends on both electron density and atomic number.^6^ For example, graphite has a large electron density; however, because its small atomic number leads to a small CT number, its SPR value deviates considerably from the HU‐SPR curve.^6^ Thus, it is necessary to replace the SPR value derived from the HU‐SPR curve with an accurate value.

For head and neck (HN) treatment in radiation therapy, the mouthpiece has been used to reduce setup errors, such as by enhancing positioning accuracy, improving inter‐fractional reproducibility, and alleviating the dose of the tongue.^7^, ^8^, ^9^ In a CT image, however, it is difficult to accurately distinguish the teeth from the mouthpiece, and it is impractical and time‐consuming to accurately contour the mouthpiece on CT images slice by slice to replace its value with an accurate SPR value. Furthermore, because the physical thickness of the mouthpiece is not large, it is believed that the error is included in the error on the HU‐SPR calibration curve or the expected beam range error considered in the margin of the target volume. However, it has recently been reported that the mouthpiece has an error of up to 13% with the HU‐SPR calibration curve, depending on the material.^9^

The East Japan Heavy Ion Center (EJHIC), located in Yamagata University's Faculty of Medicine, is the 7^th^ such facility in Japan and started treatment with carbon ions in February 2021. In preparing HN treatment, the EJHIC decided to fabricate the mouthpiece using a sample from ERKODENT, Germany, which has been used for X‐ray radiotherapy at Yamagata University Hospital. Nonetheless, for ERKODENT mouthpiece samples, the error on the SPR has not yet been reported. In this study, the SPR error that could occur for the mouthpiece material used at the EJHIC was evaluated. By comparing the HU‐SPR calibration curve for the HN used at the EJHIC with the SPR value obtained by measurement and theory, the extent to which the beam range error occurred with respect to the different thicknesses of the mouthpiece used was evaluated.

## 2 MATERIALS AND METHODS

### 2.1 Theory

The stoichiometric calibration method used in this study has been described in detail by Schneider et al.^1^ and Kanematsu et al.^2^; hence, only the necessary parts are briefly described here.

#### 2.1.1 Linear attenuation coefficient and hounsfield units

The X‐ray linear attenuation coefficient μ can be expressed using the electron density $\rho_{e}$ and atomic number *Z*,^1^, ^10^, ^11^

(1) $$\mu \;=\;\rho_{e}\left({\bar{K}}^{ph}{\tilde{Z}}^{3.62}+{\bar{K}}^{coh}{\hat{Z}}^{1.86}+{\bar{K}}^{KN}\right),$$

where ${\bar{K}}^{ph}$, ${\bar{K}}^{coh}$, and ${\bar{K}}^{KN}$ are the coefficients weighted with an X‐ray energy spectrum for the photoelectric effect, coherent scattering, and Compton scattering, respectively. These coefficients can be determined by minimizing the difference between the theoretically calculated and measured values.^1^ For a mixture, $\tilde{Z}$ and $\hat{Z}$ of the effective atomic number in Equation (1) can be calculated using the weighted sum of the fraction of electrons to each atom.^1^, ^2^

The Hounsfield units can be scaled by the difference between μ of the water *w* and μ of the medium *m*,

(2) $$HU\;=\;1000\;\times \frac{\mu_{m}-\mu_{w}}{\mu_{w}}.$$

#### 2.1.2 Stopping power ratio

The SPR of the medium relative to water is calculated using the Bethe–Bloch formula and can be approximated as^1^, ^12^, ^13^

(3) $$SPR_{w}=\frac{{\left(dE/dx\right)}_{m}}{{\left(dE/dx\right)}_{w}}\approx \;\frac{\rho_{e,m}}{\rho_{e,w}}\times \frac{\ln\left(\frac{2m_{e}c^{2}\beta^{2}}{I_{m}\left(1-\beta^{2}\right)}\right)-\beta^{2}}{\ln\left(\frac{2m_{e}c^{2}\beta^{2}}{I_{w}\left(1-\beta^{2}\right)}\right)-\beta^{2}},$$

where $m_{e}$ and *c* are the electron mass and velocity of light, β is the velocity of the ion relative to *c*, and $I_{m}$ and $I_{w}$ are the mean excitation potentials of the medium and water, respectively, in which the Bragg additivity rule can be applied to the mixture.^14^

In this paper, the SPR is considered to have the same notation as $SPR_{w}$. As shown in Equation (3), the SPR depends on the kinetic energy of the ions. If the medium is a mixture, dE/dx of the mixture can be evaluated according to Bragg's Rule,^15^

(4) $$\frac{1}{\rho }\frac{dE}{dx}\;=\;\frac{w_{1}}{\rho_{1}}{\left(\frac{dE}{dx}\right)}_{1}+\;\frac{w_{2}}{\rho_{2}}{\left(\frac{dE}{dx}\right)}_{2}\;+\;\cdots ,$$

where ρ is the mass density, and *w*_1_, *w*_2_, and so forth are the fractions by weight of medium 1, 2, and so forth.

Water equivalent length (WEL) or water equivalent path length (WEPL) is the ratio of the water‐equivalent thickness of the material to the thickness of the material, which is approximated using $\bar{{\left(dE/dx\right)}_{m}}/\bar{{\left(dE/dx\right)}_{w}}$, such that the WEL is approximated with the SPR (WEL≈SPR).^16^ As explained by Jäkel et al., the WEL can be calculated from the ratio of ΔR and the physical thickness of the medium $t_{m}$, where ΔR is the difference between the range *R* passing through only water and the range passing through the medium in front of the water.^17^

(5) $$WEL\;=\;\frac{\Delta R}{t_{m}}.$$

### 2.2 Mouthpiece

Two samples, a single‐layer sample of Erkoflex and a double‐layer sample of Erkoloc‐pro, fabricated by ERKODENT, Germany, were chosen as materials for the mouthpiece at the EJHIC. These samples were used for the X‐ray treatment of the HN at Yamagata University Hospital. Both samples were 3 mm thick and 120 mm in diameter. Table 1 lists the characteristics of the mouthpieces. Because the chemical formulations do not have exact information provided by the manufacturer, they were estimated by a simple arithmetical summation from the material information of the samples, which were used to calculate the theoretical value using Equations (1) and (3). The dental technician at our hospital constructed the shape of the mouthpiece with a sample of Erkoloc‐pro and gently wrapped the outer part with a sample of Erkoflex to produce a mouthpiece with a thickness of approximately 5 mm or less. Therefore, a combination of the two samples, Erkoflex+Erkoloc‐pro, was also considered for evaluation.

**TABLE 1:** Characteristics of mouthpiece samples provided by manufacturer.

Sample	Layer	Composition	Estimated chemical formulation	Density (g/cm)	Thickness (mm)
Erkoflex	Single layer	EVA	C_6_H_10_O_2_	0.944	3
Erkoloc‐pro	Soft layer	TPU	C_4_H_4_O_4_	1.06	3
	Hard layer	PET‐G	C_4_H_6_O_4_N_2_	1.27	

Abbreviations: EVA, ethylene‐vinyl acetate; TPU, thermoplastic polyurethane; PET‐G, polyethylene terephthalate.

### 2.3 Theoretical values of CT number and SPR

**FIGURE 1:**
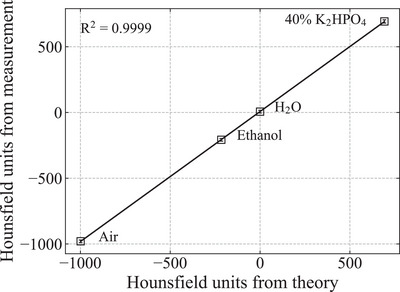
Correlation between the theoretical values of CT number calculated using Equation (1) and (2) and the measured values of CT number. Using Equation (1), ${\bar{K}}^{ph}$ = 1.9 $\times 10^{-5}$, ${\bar{K}}^{coh}$ = 1.0 $\times 10^{-3}$, and ${\bar{K}}^{KN}$ = 0.9 were found. The standard deviation (1σ) of the measured values is also displayed.

The theoretical values of the CT number of the two samples described in Section 2.2 and the combination of samples Erkoflex+Erkoloc‐pro were calculated using Equations (1) and (2). To solve Equation (1), it is necessary to determine ${\bar{K}}^{ph}$, ${\bar{K}}^{coh}$, and ${\bar{K}}^{KN}$. Following these methods,^1^, ^2^ the CT numbers in air, ethanol, water, and 40% K_2_HPO_4_ were measured according to the HN protocol with a therapeutic CT scanner (Aquilion ONE, Canon Medical Systems) used at the EJHIC.^18^ ${\bar{K}}^{ph}$ = 1.9$\;\times 10^{-5}$, ${\bar{K}}^{coh}$ = 1.0 $\times 10^{-3}$, and ${\bar{K}}^{KN}$ = 0.9 were determined, in which the difference between the measured CT number and the theoretical value calculated using Equations (1) and (2) was minimized (see Figure 1).

**FIGURE 2:**
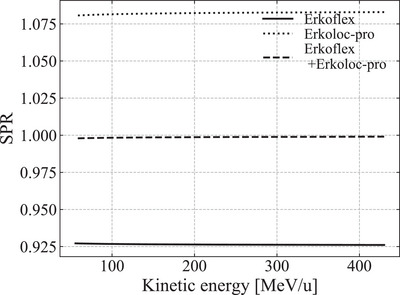
SPR calculated using Equation (3) for Erkoflex, Erkoloc‐pro, and Erkoflex+Erkoloc‐pro against the therapeutic energy of carbon ions available at the EJHIC.

The theoretical SPR values of the aforementioned samples were calculated using Equation (3). Because Equation (3) depends on the kinetic energy of the particles, it is necessary to evaluate its dependence on the therapeutic energy at the EJHIC (see Figure 2). For each sample, the theoretical values of the atomic mass, mass density, and mean excitation potential of each atom were obtained from the Particle Data Group.^19^

### 2.4 Measured values of CT number and WEL

Each sample introduced in Section 2.2 was scanned using the HN protocol with the therapeutic CT mentioned in Section 2.3. The HN protocols at the EJHIC are as follows: field of view = 500 mm, tube voltage = 120 kV, tube current = 250 mA, scan slice thickness = 0.5 mm, reconstruction slice thickness = 2 mm, and reconstruction kernel = FC13. To simulate a real patient, each sample was independently sandwiched between CIRS anthropomorphic head phantoms, and the phantoms were placed perpendicular to the scan direction. The CT images scanned for each sample were transmitted to the commercial treatment planning system (TPS) RayStation, version 10A (RaySearch Laboratories, Sweden) at the EJHIC, and the mean value and standard deviation of the CT number at each sample size were obtained.

To calculate the WEL of each sample, following the methods,^17^, ^20^, ^21^ the integral depth dose (IDD) near the Bragg peak was measured using an ionization chamber with concentric electrodes (ICCE) contained in a water tank at the horizontal port of the EJHIC. The IDDs of the Bragg curve were measured with no sample and when a sample was placed vertically in front of the water tank. As mentioned in Section 2.3, because the SPR is slightly dependent on the kinetic energy of ions, an un‐scanned carbon‐ion pencil beam with three therapeutic energies of 292.1, 180.9, and 118.8 MeV/u was irradiated; these are thought to pass through the mouthpiece in HN treatment. For each carbon ion energy, the theoretically predicted ranges in water were 164.1, 72.9, and 34.9 mm, respectively.

To obtain ΔR in Equation (5) from the measured Bragg curves, two methods were used: obtaining ΔR via Gaussian fitting of the Bragg peak, and finding ΔR by minimizing the root‐mean‐square error by shifting the Bragg curve. The difference in ΔR between the two methods was less than 1%. The WEL was then found by averaging the ΔR calculated using each method. The thickness of the sample required in Equation (5) was averaged by measuring the thickness five times at the position where the beam passed.

### 2.5 HU‐SPR calibration curve

The CT calibration method introduced by Kanematsu et al. is recommended under the name J‐CROS at carbon ion facilities in Japan,^18^, ^22^ and this method has also been adopted at the EJHIC. Following the method, a cylinder of 2 cm in diameter was filled with air, ethanol, water, and 40% K_2_HPO_4_ and inserted into a cylindrical phantom of 20 cm in diameter.^23^ CT was performed according to the EJHIC HN protocol described in Sections 2.3 and 2.4, such that the HU‐SPR calibration curve was created by averaging the measured CT number.^18^ The relative error between the SPR derived from the CT number through the HU‐SPR calibration curve (CT‐derived SPR) and the values determined in Sections 2.3 and 2.4 was calculated using the following equation:

(6) $$\begin{array}{c} Relative\;error\;\left[\%\right]=\left(Value/CT\;derived\;SPR\right)\times 100. \end{array}$$

### 2.6 Comparison of clinical dose distribution

A sample of Erkoflex+Erkoloc‐pro was vertically attached to the side of an ISIS Phantom Cube (RPD Inc., Albertville, Minnesota, USA), which is a 14 cm acrylic cube, and was then CT‐scanned at the EJHIC (mentioned in Section 2.3) according to the HN protocol. The scanned CT images were imported into the RayStation TPS.

A 6 × 6 × 6 cm^3^ target was defined at the center of the ISIS Phantom Cube. Because the ISIS Phantom Cube contained various materials, the SPR of the entire ISIS Phantom Cube was manually set to water. A uniform relative biological effectiveness (RBE)‐weighted dose, hereafter referred to as the clinical dose,^24^ of 4 Gy (RBE) passing through Erkoflex+Erkoloc‐pro and ISIS Phantom Cube was planned for the target with scanned carbon‐ion beams. The spot spacing and energy slice spacing were set to 2 and 1.5 mm, respectively. The number of ions for each spot was determined through the TPS optimization process to cover at least 95% of the clinical dose covering the entire target. A biological microdosimetric kinetic model (MKM) was utilized to calculate the clinical dose.^25^

Two dose distributions were created: 1. A calculated clinical dose distribution after converting the CT number to SPR using the HU‐SPR calibration curve of the HN protocol (CT‐derived SPR), 2. A determined clinical dose distribution from the corresponding beam information (beam energies, spot weight, and position) of the calculated dose distribution, after only a portion of the sample of Erkoflex+Erkoloc‐pro was converted to a WEL value of 1.060 (see Table 3). The displacement of the two dose distributions was then calculated by the difference at the depth corresponding to 50% height of the clinical dose at the distal fall‐off region.

## 3 RESULTS AND DISCUSSION

**FIGURE 3:**
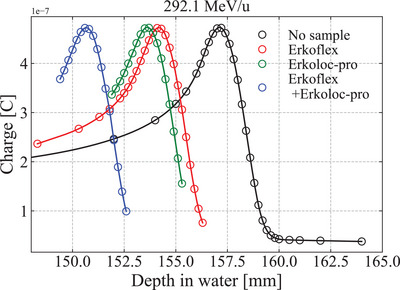
Measured Bragg curves of carbon ions of 292.1 MeV/u in water using an ICCE with no sample and when a sample is placed vertically in front of the water tank. Each solid line is a line calculated using the spline interpolation method.

Figure 1 shows the correlation between the theoretical and measured values of the CT number when ${\bar{K}}^{ph}$ = 1.9 $\times 10^{-5}$, ${\bar{K}}^{coh}$ = 1.0 $\times 10^{-3}$, and ${\bar{K}}^{KN}$ = 0.9 were used. Figure 2 shows the SPR calculated using Equation (3) for Erkoflex, Erkoloc‐pro, and Erkoflex+Erkoloc‐pro against the therapeutic energy of the EJHIC. Although the SPR fluctuated slightly at low kinetic energies, it was confirmed that the SPR for the therapeutic energy at the EJHIC varied by less than 0.1% for 1σ. Figure 3 shows the measured Bragg curve of carbon ions of 292.1 MeV/u in water using an ICCE when no sample was used and the Bragg curves of carbon ions of 292.1 MeV/u when a sample was placed vertically in front of the water tank.

**FIGURE 4:**
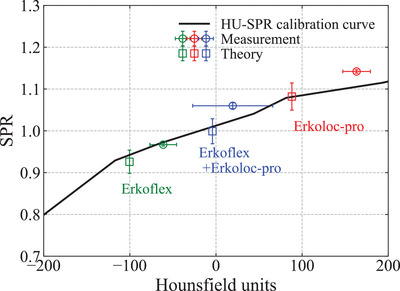
HU‐SPR conversion curve for the HN protocol at the EJHIC obtained using the method described in Section 2.5, and the values of the CT number and SPR of each sample calculated and measured using the method described in Sections 2.3 and 2.4. In the case of the measured value, the standard deviation is displayed, and in the case of the theoretical value of the SPR, an error of 3% is presented.

Figure 4 shows the HU‐SPR calibration curve for the HN protocol at the EJHIC obtained using the method described in Section 2.5, and the values for each sample were calculated and measured using the method described in Sections 2.3 and 2.4. For each sample, the theoretical and experimental values and the error with the HU‐SPR calibration curve are shown in Tables 2 and 3, respectively. Regarding the measured CT number, because Erkoflex and Erkoloc‐pro were overlaid, the standard deviation of the CT value of Erkoflex+Erkoloc‐pro shown in Figure 4 was relatively large.

**TABLE 2:** Theoretically calculated values of the CT number and SPR of the samples, and SPR values from the HU‐SPR calibration curve (CT‐derived SPR) and the error on the SPR with the CT‐derived SPR.

Sample	CT number from theory	SPR from theory	CT‐derived SPR	Relative error on SPR [%]
Erkoflex	−100.44	0.926	0.942	−1.7
Erkoloc‐pro	88.09	1.082	1.081	0.1
Erkoflex+ Erkoloc‐pro	−4.09	0.999	1.010	−1.1

Abbreviations: SPR, stopping power ratio; HU, Hounsfield units.

**TABLE 3:** Mean values of the measured CT number and WEL of the samples, the SPR values from the HU‐SPR calibration curve (CT‐derived SPR), and the error on the SPR with the CT‐derived SPR.

Sample	CT number from measurement	WEL from measurement	CT‐derived SPR	Relative error on SPR [%]
Erkoflex	−61.25	0.967	0.973	−0.5
Erkoloc‐pro	163.23	1.142	1.105	3.3
Erkoflex+ Erkoloc‐pro	19.43	1.060	1.025	3.4

Abbreviations: SPR, stopping power ratio; HU, Hounsfield units; WEL, water equivalent length.

As shown in Figure 4, the measured value of the CT number was greater than the theoretical value of the CT number for each sample. Comparing the error between the measured μ value calculated using Equation (2) and the theoretical μ values of Erkoflex, Erkoloc‐pro, and Erkoflex+Erkoloc‐pro, the measured values were 4.4%, 6.9%, and 2.4% greater than the theoretical value, respectively. It is possible that an error in the CT value occurred because of the small thickness of the sample used for the measurement (3 mm). As reported by Schaffner et al.,^26^ because CT X‐rays are not of a single energy, a variation of 5% in CT values may occur depending on the size of the phantom or the location of the sample. However, this error is thought to be a probable error that can occur even on the CT image of a patient because the fabricated mouthpiece is also thin. Although the theoretically calculated CT number was closer to the HU‐SPR calibration curve, it is thought that the error in the CT number, as shown in Figure 4, will occur in actual CT number measurements. The aforementioned differences of 4.4%, 6.9%, and 2.4%, between the measured and theoretical μ values of Erkoflex, Erkoloc‐pro, and Erkoflex+Erkoloc‐pro, respectively, resulted in a difference of 3.3%, 2.2%, and 1.5%, respectively, in the CT‐derived SPR (errors in the fourth column between Tables 2 and 3). The μ value depends on the atomic number and electron density, as shown in Equation (1), whereas SPR depends on the electron density, as shown in Equation (3). Thus, as the atomic number increases, the μ value is affected more than the SPR because of the effect of the square of the atomic number, such that the slope of the HU‐SPR conversion curve becomes smaller. Therefore, the Erkoloc‐pro sample was considered to have a relatively small error of 2.2% in the CT‐derived SPR, while it had a large error of 6.9% from the theoretical value in the μ value.

We found that the theoretical values of SPR and WEL differed by up to 5.8%. The cause for this error was unclear, but presumably due to errors from differences in the estimated and actual material composition and density of the sample provided by the manufacturer and errors in the Bragg rule calculation and mean excitation potential.^6^ Compared against the HU‐SPR calibration curve, the CT‐derived SPR from the theoretical value exhibited an error within 1.8%, and the measured WEL value exhibited an error within 3.5% from the CT‐derived SPR from the measured CT number. The 0.5% error on the CT‐derived SPR from the measured CT number for Erkoflex was similar to the CT‐derived SPR error of 0.7−2.3% for the ethylene‐vinyl acetate (EVA) material reported by Ikawa et al.^9^ They reported that EVA had the lowest SPR error out of the materials tested because EVA is the material most similar to tissue composition. An error of 3.5% of the CT‐derived SPR value from the WEL value may be further reduced if a dual‐energy CT or photon‐counting CT is utilized. According to Yang et al.,^27^ the maximum error in SPR reduced from 3.24% to 1% when using dual‐energy CT compared with using a single HU‐SPR conversion curve for human biological tissues. Boruque et al.^28^ reported a mean absolute error of 0.5 ± 0.4% for SPR calculated with dual‐energy CT for human tissue properties. Furthermore, according to a recent experimental report by Simard et al.,^29^ the use of photon‐counting CT on the Gammex phantom reduced the root‐mean‐square error of SPR from 1.92% to 0.89% compared with the use of dual‐energy CT. Their study showed that photon‐counting CT was slightly superior to dual‐energy CT. In the case of photon‐counting CT, because the CT image was reconstructed by dividing the energy bins of the X‐ray spectrum, it had the advantage of being less affected by the beam hardening effect compared with the single‐energy CT.^6^, ^30^ Alternatively, it may also be useful to calculate a target margin by considering the error of the HU‐SPR conversion curve using a robust optimization method in TPS.^31^

**FIGURE 5:**
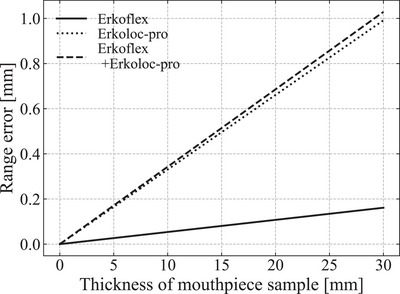
Estimated beam range error according to the increase in the thickness of each sample, calculated from the errors between the values of the CT‐derived SPR and the WEL values shown in Figure 4 and Table 3.

Figure 5 shows the estimated beam range error according to the increase in the thickness of each sample, calculated from the errors between the CT‐derived SPR and WEL values shown in Figure 4 and Table 3. Because the thickness of the mouthpiece manufactured at the EJHIC is approximately 5 mm on one side, when the beam passes through the 10‐mm thick mouthpiece (Erkoflex+Erkoloc‐pro), including the other side, the beam range error is expected to be approximately 0.4 mm, as shown in Figure 5. However, in the portion of the mouthpiece that covers the top of the teeth, the beam passes through the mouthpiece as thick as the teeth. In this case, it is thought that the beam passes through approximately 30 mm of the mouthpiece, and there is a possibility that a beam range error of approximately 1 mm may occur. If this error is not considered, the beam will stop at a point 1 mm shallower than expected. In CT images, it is difficult to accurately distinguish the mouthpiece from the CT image because the teeth and mouthpiece are in close contact and the CT number of the teeth is high. Thus, to apply this error in the patient dose calculation, it may be more practical to add a tumor margin than to accurately contour a mouthpiece and replace its CT‐derived SPR with the measured WEL value.

**FIGURE 6:**
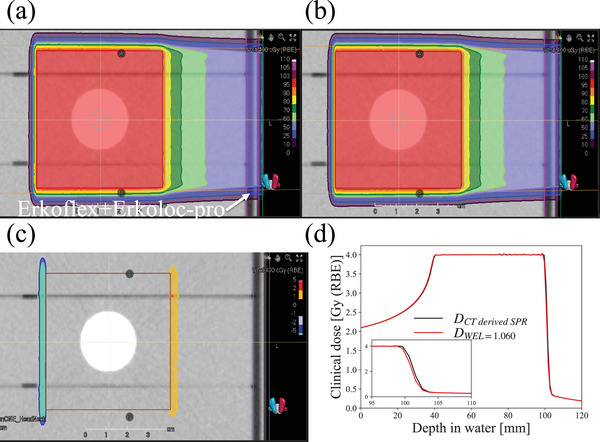
(a) Calculated clinical dose distribution by the SPR converted from the HU‐SPR calibration curve of the HN protocol ( = CT‐derived SPR), (b) determined clinical dose distribution from the beam energies, and the spot weights and positions corresponding to (a) after only a portion of the sample of Erkoflex+Erkoloc‐pro (part contoured in purple color) was converted to a WEL value of 1.06, (c) difference in clinical dose distributions of (a) and (b) (b‐a), (d) clinical depth dose distribution of (a), $D_{CT\;derived\;SPR}$, and (b), $D_{WEL\;=\;1.060}$, at the middle position of the ISIS Phantom Cube. The inset in (d) is an enlarged image of the clinical dose distribution at the distal region.

Figure 6 shows the difference between the calculated and determined clinical dose distributions mentioned in Section 2.6. A displacement of 0.23 mm of the clinical dose distribution was found from the difference between the dose distributions by the CT‐derived SPR and that by the WEL value for a 6‐mm thick Erkoflex+Erkoloc‐pro (Figure 6d). This result is in close agreement with the results shown in Figure 5. Even with a displacement of 0.23 mm for the dose distribution, hot and cold spots are clearly visible in Figure 6c.

## 4 CONCLUSION

We calculated the SPR error for a mouthpiece sample from ERKODENT selected at the EJHIC. Each sample was CT scanned using the HN protocol from the EJHIC, and the values of the sample area were averaged to obtain the CT number. In addition, Bragg curves passing through each sample were measured for three energies at the horizontal port of the EJHIC, and the WELs were obtained by averaging them. To calculate the difference between the theoretical and measured values, the theoretical CT number and SPR value of the samples were calculated using the stoichiometric calibration method by determining the three coefficient values necessary for calculating the linear attenuation coefficient. The WEL value of the mouthpiece sample had an error of approximately 3.5% from the CT‐derived SPR from the measured CT number. Using this error, it was evaluated that for a mouthpiece with a thickness of 10 mm, a beam range error of approximately 0.4 mm can occur, and for a mouthpiece with a thickness of 30 mm, a beam range error of approximately 1 mm can occur. For a beam passing through the mouthpiece in HN treatment, it would be practical to consider a mouthpiece margin of 1 mm to avoid beam range errors.

## AUTHOR CONTRIBUTIONS

Sung Hyun Lee−preparation, creation and presentation of the published work; Takayuki Kanai−formulation or evolution of overarching research goals and aims; Hikaru Souda−conducting a research and investigation process; Yuya Miyasaka−development or design of methodology; Hongbo Chai−provision of study materials, reagents, materials; Takuya Ono−verification; Yoshifumi Yamazawa−data curation; Koji Suzuki−oversight and leadership responsibility for the research activity planning and execution; Azusa Sato and Masashi Katsumata−performing the experiments; Takeo Iwai−management and coordination responsibility for the research activity planning and execution. All authors have agreed to the published version of the manuscript.

## CONFLICT OF INTEREST

No conflict of interest.

## Data Availability

The data that support the findings of this study are available from the corresponding author upon reasonable request.
